# Learning (from) the errors of a systems biology model

**DOI:** 10.1038/srep20772

**Published:** 2016-02-11

**Authors:** Benjamin Engelhardt, Holger Frőhlich, Maik Kschischo

**Affiliations:** 1 Rheinische Friedrich-Wilhelms-Universität Bonn, Institute for Computer Science, Algorithmic Bioinformatics, c/o Bonn-Aachen International Center for IT, Dahlmannstr. 2, 53113, Bonn, Germany; 2 University of Applied Sciences Koblenz, RheinAhrCampus, Department of Mathematics and Technology, Joseph-Rovan-Allee 2, 53424 Remagen, Germany

## Abstract

Mathematical modelling is a labour intensive process involving several iterations of testing on real data and manual model modifications. In biology, the domain knowledge guiding model development is in many cases itself incomplete and uncertain. A major problem in this context is that biological systems are open. Missed or unknown external influences as well as erroneous interactions in the model could thus lead to severely misleading results. Here we introduce the dynamic elastic-net, a data driven mathematical method which automatically detects such model errors in ordinary differential equation (ODE) models. We demonstrate for real and simulated data, how the dynamic elastic-net approach can be used to automatically (i) reconstruct the error signal, (ii) identify the target variables of model error, and (iii) reconstruct the true system state even for incomplete or preliminary models. Our work provides a systematic computational method facilitating modelling of open biological systems under uncertain knowledge.

Mathematical models of living systems are increasingly used in systems biology to gain important biological insights and to make testable predictions^1^,^2^,^3^. Ideally, a good model covers the essential features of the system whilst still being simple enough for interpretation and mechanistic understanding. Developing a good model is usually a labour intensive manual effort. In biology, the system to be modelled is often only partially known and the distinction of relevant and irrelevant features and variables can be difficult^4^,^5^,^6^,^7^,^8^. But, even if the major components of a biological system are well known, the sheer complexity of the system might prevent the development of an accurate mathematical model, either because the quantitative data necessary for modelling are not available or because the model is itself too complex to be useful. Thus, researchers in systems biology are frequently confronted with a paradoxical situation: A model is needed to better understand the system and to design informative experiments, but the system is too large and complex for mathematical modelling given the limited amount of knowledge, data and time.

One strategy for modelling is to start with a simple model, which incorporates the most interesting variables and interactions as well as the known input stimuli to the system (Fig. 1a). For example, to model a biochemical reaction network, we might incorporate the concentrations of a few interesting proteins as dynamic state variables and integrate the knowledge about the reactions into simplified assumptions about the interactions between these states. We refer to this simple draft model as the nominal system.

There are two reasons, why the nominal model might not be in sufficient agreement with the experimental data (Fig. 1b): First, some interactions between the nominal state variables could be missing or misspecified. For a reaction network that means there are missing biochemical reactions, incorrect assumptions about the reaction kinetics or inaccurate parameter estimates. Second, the nominal system is in fact—opposed to the typical situation in many areas of physics—open and embedded into a larger dynamic system^9^. Exogenous variables, which are not incorporated, but interact with the nominal model might act as hidden inputs and thereby alter the dynamics of the nominal system. It is the task of the modeller to first identify the most relevant errors in the nominal model and then compare different model versions in order to achieve a better fit to the available experimental data. This process is labour intensive and in many cases a trial and error exercise, even with the help of innovative software and algorithms assisting modelling and model comparison^5^,^6^,^7^,^10^,^11^,^12^,^13^,^14^.

Here, we introduce a computational method for ordinary differential equations (ODEs), which automatically estimates the model error from the data. ODEs are frequently used in different areas of biology including biochemical reaction networks, pharmacokinetics, pharmacodynamics and population dynamics.

The basic idea of the method is to represent errors in the nominal model as hidden inputs to the state variables (Fig. 1c) and to estimate these inputs from the experimental data^8^,^15^,^16^,^17^. Since this is an inverse problem with potentially many different solutions, we propose a regularised method which provides parsimonious error estimates. Due to its formal similarity to the elastic-net regression approach^18^, we term our algorithm the dynamic elastic-net.

The dynamic elastic-net provides important information about the variables in the nominal model, which are targeted by model errors. In addition, the dynamic elastic-net removes the bias in the nominal state variables induced by the model error. This is important for the frequent situation, that not all nominal states (e.g. protein concentrations) can directly be measured. The utility of the dynamic elastic-net is demonstrated here for two established models of the EPO receptor^4^ and of the photomorphogenic UV-B signalling network^19^. Further examples including a model for G protein signalling and models for several network motifs as well as some technical details are given in Supplementary text.

## Results

### The nominal model

**Figure 1:**
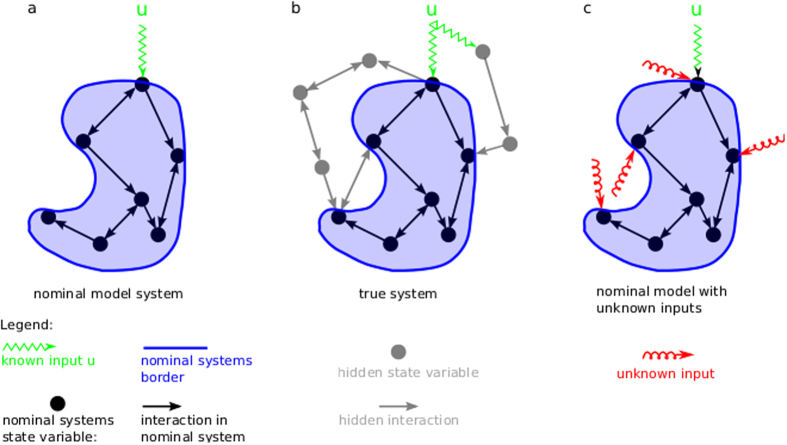
(**a**) The nominal model represents the current assumptions about the true system. The systems model is specified by its dynamic state variables and their interactions, here represented as vertices and edges of a graph. The systems border defines the distinction between internal states and exogenous inputs. The exogenous inputs *u* are assumed to be known. (**b**) In reality, the nominal model is embedded in a larger network outside the nominal systems border. The hidden dynamics of the exosystem interacts with the nominal system. In addition, some interactions between nominal state variables might be missing or misspecified in the nominal model. These model errors can potentially lead to discrepancies between model and experimental data. (**c**) Representation of model errors as hidden inputs to the nominal model. The dynamic elastic-net approach infers the hidden inputs from data and thereby corrects for the bias in the nominal state variables induced by model errors.

We assume that a nominal ODE model


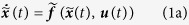



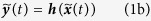



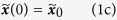


has been proposed to describe the dynamics of the system of consideration. The state vector 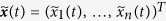 contains the *n* dynamic variables 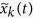, and 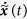 is the derivative with respect to time *t*. The initial value of the state vector is 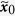. For a biochemical reaction network, 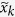 is often the concentration or abundance of the *k*-th species. The function 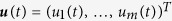 represents a known external input to the system. The dynamics of the state variables is determined by the function 
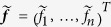
 and encodes the model assumptions made in the nominal model. This can be represented as a graph^20^, where each node corresponds to one variable and a directed edge from *l* to *k* indicates, that the time derivative of 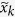 depends on 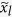 (Fig. 1a). If 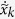 is directly influenced by a known input, we illustrate this by a green zigzag arrow. Typically, not all state variables 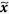 can directly be measured. The variables 
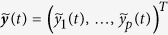
 represent all outputs which are experimentally accessible. In equation (1b), we assume that the mapping ***h*** from the state ***x*** to the output ***y*** is known. We use a tilde to highlight, that 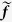 and thus 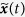 are usually not perfectly known due to limited or uncertain knowledge about the true underlying dynamics.

### Representation of the model error

The response of the real natural system to a known input stimulus 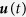 is usually measured at discrete time points 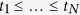 and provides experimental observations for the output 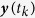. A part of these data is usually used to estimate the parameters of the model. We consider the initial parameter estimates as part of the nominal model specification 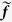 in equation (1a).

The nominal model is unsatisfactory, when its output 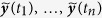 is not in sufficient agreement with the data 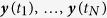. One source of model error comes from hidden inputs to the nominal system, which are caused by dynamical processes exogenous to the nominal system (Fig. 1b). In addition, there might be missing or erroneous interactions between the state variables 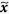 in the nominal model itself. Both types of model error can be represented by hidden inputs 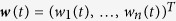 acting on the nodes of the nominal model (Fig. 1c). The “true” dynamics 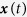 of the real system can be described by


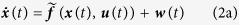



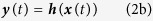



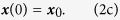


Here, the state 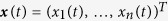 represents the same variables as the nominal state 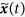, but we suppress the tilde to distinguish solutions of (2) from that of the the nominal model. The model error is the difference 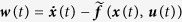 between the rate of change of the true system 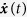 and the nominal system 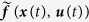, evaluated along the true state trajectory 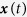. Thus, it incorporates any discrepancy between the true system and the nominal system. The known input ***u*** and the output function ***h*** are assumed to be identical to the nominal model (1). However, we will also discuss the impact of measurement noise below.

The typical approach to model improvement is to compensate for the model error 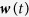 by explicit mathematical expressions, often additional differential equations. This increases the number of variables and parameters in the model. Here, we proceed differently by estimating the model error ***w*** from the data, what also enables us to correct for the bias 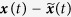 of the state estimate incurred by the nominal model.

### Estimating the unmodelled dynamics

To estimate the model error 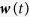, we use the observer system


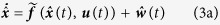



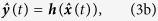


which is a copy of equations (2a) and (2b). The hat marks estimates of the state 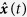, of the output 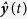 and of the model error 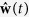. The latter is obtained by minimising the error functional


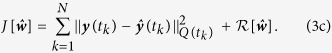


The first term in equation (3c) is the weighted mean square error between the measured outputs 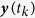 and the outputs 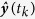 of the observer system in equations (3a) and (3b). The weighted square norm


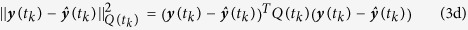


contains the symmetric weighting matrix 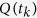, which is often chosen to be diagonal and can be used to transform outputs of very different magnitude to a common scale or to incorporate precision estimates of the measurements at the different time points 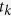. The regularisation term


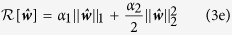



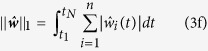



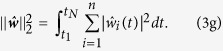


is necessary to avoid overfitting of the data 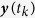 by overly complex estimates 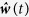. The nonnegative parameters 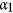 and 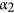 determine the relative contributions of the 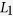 norm in equation (3f) and of the 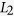 norm in (3g). Minimisation of equation (3c) under the constraints in equations (3a) and (3b) is an optimal control problem^21^,^22^,^23^, which needs be solved numerically (see Methods and Supplementary Text).

The combined 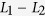 regularisation in equation (3e) is reminiscent of the elastic-net penalty used in regression models^18^. Therefore, we termed our approach the dynamic elastic-net. In analogy to regression, the 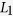 term causes some components 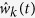 of the estimated model error to shrink to zero (Supplementary text). The amount of shrinkage is determined by 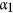, which can be chosen to suppress small error signals or noise distributed over many components of the estimate 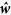. The resulting sparse estimate is useful, because it provides information about the states of the system which are targeted by systematic model errors, as represented by hidden inputs.

In contrast to regression, a pure 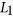 or Lasso type^24^ regularisation is not useful in the dynamic setting, because the solution for 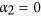 can result in unbounded estimates of 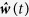. Even when additional constraints on 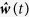 are imposed, the resulting solution is not smooth and either zero or at the boundaries of the constraints^25^. These insights about the optimal control problem can be obtained from Pontryagin’s minimum principle^21^,^22^, as it is detailed in the Supplementary text together with some strategies to chose suitable regularisation parameters 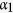 and 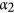. In addition to sparse but smooth estimates of the model error, the dynamic elastic-net automatically provides a state estimate 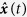. Often this is very interesting information, when not all state variables are experimentally accessible.

The optimal control problem in equations (3a–c) for 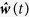 requires the specification of an initial condition 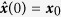, which is often not known or uncertain. Alternatively, one can add the additional constraint


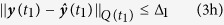


to (3a–c), where 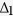 is a preset tolerance given for the fit of 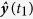 to 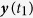 at time 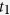. Similarly, a tolerance 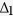 can be prescribed to the fit at the last data point by


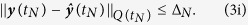


The tolerance parameters 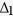 and 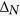 of these optional constraints can often be obtained from error bars of the measurements.

### Validation of the dynamic elastic-net

#### JAK-STAT signalling example

**Figure 2:**
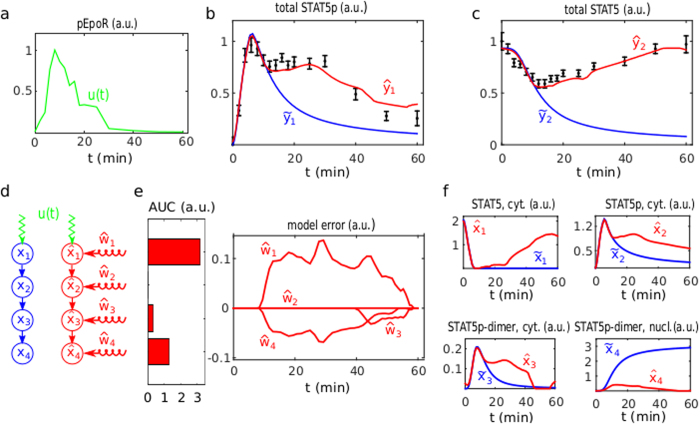
Estimating the model error for the JAK-STAT pathway. (**a**) The known input 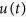 is given by linearly interpolated phosphorylation measurements for the erythropoietin receptor^4^. (**b**,**c**) The output measurements^4^ (black) for phosphorylated STAT5 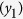 and total STAT5 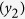 in the cytoplasm compared to the outputs of the nominal model (blue) and the fit of the dynamic elastic-net (red). (**d**) Graph of the nominal model (blue) and of the observer system (red) with the state variables cytoplasmatic STAT5 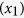, phosphorylated monomeric STAT5 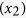, phosphorylated dimeric STAT5 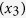 and nuclear STAT5 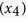. (**e**) Dynamic elastic-net estimates 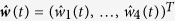 of the model error and the area under the curve (AUC) for the magnitude of each component 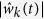. (**f**) The state estimates 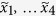 obtained from the nominal model (blue) and the dynamic elastic-net observer (see 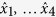 in red).

To illustrate the dynamic elastic-net estimator for a small and comprehensible model we used established experimental data for the JAK-STAT signal transduction pathway^4^. The four state variables of the system represent unphosporylated cytoplasmatic STAT5 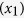, phosphorylated monomeric STAT5 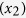, phosphorylated dimeric STAT5 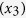 and nuclear dimeric STAT5 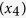. The nominal model^4^


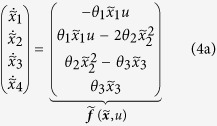



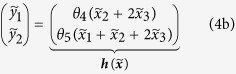


describes the phosphorylation of cytoplasmatic STAT5 upon activation of the erythropoietin receptor (known input *u*), the dimerisation of phosphorylated STAT5 and the export to the nucleus (Fig. 2). Time course data^4^ for the amount of cytoplasmatic phosphorylated STAT5 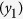 and total cytoplasmatic STAT5 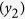 were used to calibrate the parameters 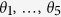. However, the presence of systematic model error is apparent from the inalterable discrepancy between the experimental data and the nominal model incorporating optimised parameter values (Fig. 2b,c).

To estimate this model error 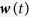, we numerically fitted the dynamic elastic-net (3) with the nominal model (4) to the output measurements. To quantify the magnitude of the different components, we numerically computed the area under the curve (AUC) of each 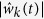, i.e. 
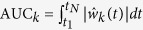
. The AUC and the estimated time course 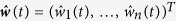 of the model error indicate (Fig. 2e), that the dominant contributions 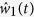 and 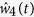 of the model error target the states 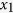 and 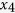, representing the amount of unphosphorylated cytosplasmatic STAT5 and nuclear STAT5. The second component 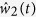 of the dynamic elastic-net estimate is identically zero for the whole time interval (Fig. 2e). Apart from the small signal 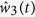 initiated after approximately 40 mins, this is consistent with the improved nucleocytoplasmatic cycling model reported in^26^, which is based on the same data^4^ and incorporates the relocation of dephosphorylated nuclear STAT5 molecules into the cytoplasm. Importantly, the dynamic elastic-net also provides modified estimates for the four STAT5 state variables (Fig. 2f), which are also in good agreement with the nucleocytoplasmatic cycling model (Supplementary Text).

An important problem with regularisation approaches is the choice of the regularisation parameters 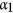 and 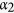. We used 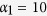 and 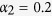 in Fig. 2, but we found empirically, that the AUC values clearly indicate the target points of the model error for a wide range of 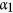-values (Supplementary Fig. S2). The 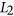 parameter 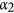 was chosen to balance the smoothness of 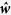 and the accuracy of the fit to the output measurements. In addition, the bias induced by the double regularisation^18^ can be compensated by a simple thresholding strategy: Given an initial estimate 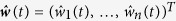 of the model error, we refit the dynamic elastic-net by constraining all the components with small AUC to zero. Thresholding is known in the regression context^27^ and we found it to improve the state estimates as well as the time course estimates of the remaining model errors (Supplementary Fig. S3).

#### The impact of measurement noise and parameter uncertainties

**Figure 3:**
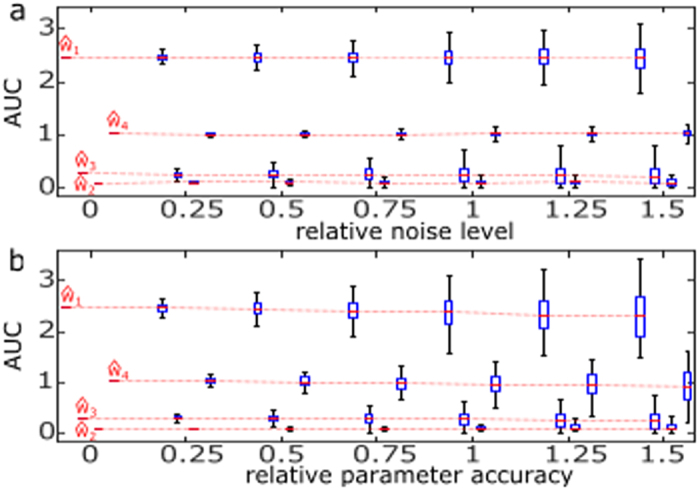
The impact of simulated measurement noise and parameter uncertainty to the dynamic elastic-net estimate in the JAK-STAT model. (**a**) Box plots visualising the variation of the AUC of 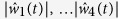 for the dynamic elastic-net estimates caused by measurement noise (see main text for details). To ease visualisation, box plots at a given noise level are slightly offset. (**b**) The variation of the AUC caused by parameter uncertainty.

To explore the robustness of the dynamic elastic-net against measurement noise, we added random perturbations to the experimental data^4^. For a given noise level, we generated 500 perturbed data sets by adding Gaussian random numbers with mean zero and standard deviation scaled by a multiple of the empirical standard deviation (see the error bars in Fig. 2b,c) to each experimental data point. Thus, the noise level is defined as a multiple of the empirical standard deviation. The dynamic elastic-net was then fitted to each output sample and the corresponding area under the curve 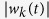 for each component of the estimated model error 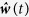 was computed. The plots for these AUC values versus the noise level are shown in Fig. 3a. The median values of the AUC for the components 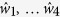 are largely independent of the noise level, but the variability of the AUC estimates increases with measurement noise. Nevertheless, the AUC values for 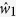 and 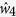 are always much larger than zero, whereas the AUC of 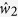 and 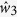 is close or even equal to zero for many samples with higher noise level. This increases the confidence that the nodes 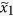 and 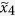 (Fig. 2d) of the nominal JAK-STAT model (4) are the main target points of the model error.

The impact of parameter uncertainty in the nominal model was assessed in a similar way. Parameter estimation algorithms^4^,^10^,^26^ applied to the nominal model using the experimental data (Fig. 2b,c) provide point estimates and confidence intervals for each component of the parameter vector. These confidence intervals were again scaled by the noise level, yielding an interval for each parameter from which uniform random samples were drawn. Again, we generated 500 modified parameter vectors per noise level. For each parameter sample, the system (4) was taken as the nominal model and the AUC of the resulting estimates 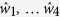 was recorded (Fig. 3b). Again, there is no systematic trend for the AUC of the different components of the estimated error 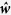. However, the variation of the AUC increases much faster than in Fig. 3a. Apart from the different sampling distributions used, this effect is related to the definition of the model error ***w***, which is always defined with respect to the nominal model (confer eqution 2a). Hence, the estimated model error 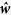 contains contributions from both structural and parameter misspecifications in the nominal model. Nevertheless, it is still possible to infer the dominant components 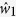 and 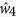 with high confidence. Similar results were found for the sensitivity against the number of measurement time points (Supplementary text, Fig. S6).

#### Photomorphogenic UV-B signalling example

**Figure 4:**
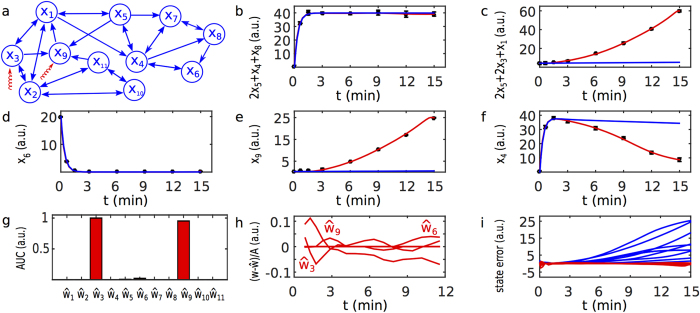
The Photomorphogenic UV-B signalling example. (**a**) The graph (without self loops) of the model states^19^. The target points of the simulated model errors are indicated by the red arrows. (**b**–**f**) The simulated output 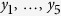 with error bars (black), the output of the nominal model (blue) and the output of the dynamic elastic-net (red). (**g**) The AUC of the absolute model errors 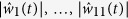. (**h**) The components of 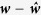 relative to the amplitude *A* of the true model error. (**i**) The discrepancy 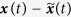 between the true state and the nominal model state (blue) compared to the discrepancy 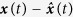 of the dynamic elastic-net (red).

As a test case for a larger system, we used a recent model for the coordination of photomorphogenic UV-B signalling in plants^19^. The model consists of 11 ODEs describing the dynamics of protein concentrations 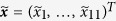 coupled by 10 chemical reactions (Fig. 4). We considered this model as the nominal model in order to test the dynamic elastic-net method for a situation, where the ground truth is known. The model error was simulated by adding the hidden inputs 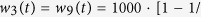

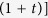
 to the nodes 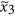 and 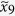. The output function 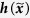 is a linear combination of 7 different state variables (see Supplementary text for all equations). Synthetic data were sampled at discrete time points from the outputs of the true model and Gaussian random perturbations were added to simulate measurement noise (Fig. 4b–f). The dynamic elastic-net with the nominal model was used to reconstruct the model error 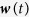 and the true state 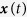 from these simulated data. The absolute area under the curve for each component of the model error estimate 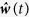 clearly indicates that the states 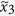 and 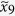 are targeted by hidden inputs (Fig. 4g), whereas all other components are either very small 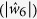 or even zero. This illustrates the sparsity of the dynamic elastic-net estimate, which is a clear advantage over pure *L*_2_ regularisation. The discrepancy 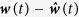 between the model error and the corresponding estimate relative to the amplitude 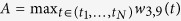 of the true model error is at most 10% (Fig. 4h) and mainly caused by numerical inaccuracies. Most importantly, the discrepancy 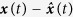 between the true and the estimated state trajectory is almost zero (Fig. 4i), indicating the excellent performance of the dynamic elastic-net as a state observer.

#### Testing the limitations

As for any inverse method, there are limitations of the dynamic elastic-net method. Some model errors 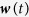 are unobservable, because there exists a different hidden input function 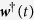 which generates an output 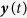 which is identical to the output obtained for 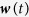, see the Supplementary text for a simple example. Other model errors might be practically unobservable, because the output for another hidden input function might not be distinguishable within the measurement errors. A special case are model errors which have no or almost no effect on the output at all. These will not be noticed during modelling and the nominal model will be accepted.

**Figure 5:**
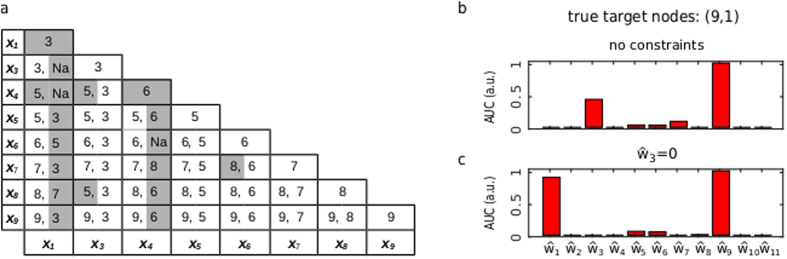
Detecting the target nodes of simulated model errors in the UV-B signalling network. (**a**) All nodes and all pairs of nodes were perturbed by a simulated model error. Nodes 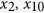 and 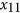 are omitted, since the simulated error signal had no effect on the output. The rows and the columns correspond to the true target nodes of the model error and the numbers in the cells are the nodes found by the dynamic elastic-net (NA means that no second node was assigned). Gray cells indicate errors made by the dynamic elastic-net for unobservable model errors. (**b**) An example for an unobservable model error. The true target nodes of the model error are 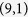, but the dynamic elastic-net predicts the target nodes 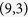. (**c**) Refitting the dynamic elastic-net under the constraint 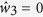 provides an alternative solution. The other two combinations 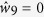 and 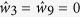 of the nodes 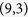 did not fit the output data.

To further test the ability of the dynamic elastic-net to infer the states targeted by the model error, i.e. the non-zero components of the true model error 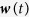, we systematically simulated perturbations to different nodes and node pairs. First, we simulated model errors 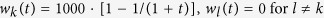 targeting a single node *k* in the same way as before. For the nodes 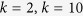 and 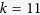 there was no effect on the output (see again Fig. 4b–f) and thus these nodes were omitted from further analysis. In addition, we simulated hidden inputs for all remaining two node combinations. For each of these 36 simulated true models we tested the ability of the dynamic elastic-net to recover the correct target nodes from the AUC of the estimated 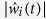. We considered a node or a node pair to be correctly recovered, if their AUC was at least 85% of the total AUC over all nodes. By this stringent criterion, we found that two single node errors targeting 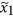 or 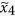 were not correctly detected and another single node was predicted to be the target of the model error (Fig. 5a). This indicates, that these model errors are unobservable and the observed output data can be explained by different inputs to different nodes. With two exceptions ((8, 3) and (7, 6)), the mistakes made by the algorithm for simulated pairwise model errors involve these two state nodes 1 and 4. However, with exception of the combination (1, 4), at least one node is correctly predicted.

These results demonstrate the inherent limitations of any attempt to recover the model error from observed outputs. For an unobservable model error, the true model error 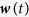 might correspond to a slightly larger value of the error functional (3c) than the minimum 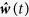 obtained by the dynamic elastic-net. A heuristic approach to explore some of these slightly suboptimal solutions is to rerun the dynamic elastic-net with some of the estimated target nodes (from the first run) excluded and to check, whether the output data can satisfactory be fitted with the same level of sparsity. This is illustrated in Fig. 5b for the node pair (9, 1), which was predicted to be (9, 3) by our criterion. Refitting the dynamic elastic under the constraint 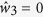 identifies the correct nodes (9, 1), see Fig. 5c. The two other combinations 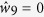 and 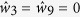 do not provide a satisfactory fit to the data (Supplemental Fig. S9). For the UVB-signaling network we find, that the slightly suboptimal solutions identified by this heuristics always contain the correct target node configuration. The combinatorial explosion of this strategy should typically not be a problem, thanks to the sparsity of the dynamic elastic-net predictions. The decision, which of the predicted target node sets, 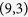 or 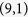, is the correct one can in practice only be made when additional states are measured. However, this example shows, how the dynamic elastic-net provides useful information to select further states for experimental observation^20^,^28^.

## Discussion

Efficient computational methods to learn from incomplete model drafts and to direct model improvement are urgently needed. Our proposed dynamic elastic-net approach provides suggestions for the location of these model errors in the network and estimates their dynamic time courses from measured output data. The sparsity of the proposed target points for the model error promotes model improvements in the most parsimonious way. Even for an incomplete nominal model the algorithm can provide estimates for the system states which are not experimentally accessible. This is in stark contrast to many other state estimators including the Kalman Filter^29^ for linear systems and its various extensions for nonlinear systems^30^,^31^, which usually require a correctly specified model.

Not all model errors can uniquely be determined from the output. For such unobservable model errors, our strategy to explore alternative, slightly suboptimal solutions might indicate alternative explanations for observed discrepancies between the data and the nominal model. In addition, this approach can also be informative for selecting additional nodes required for observing the state from output measurements^20^,^28^. Further research is needed to establish the relationship between the network topology and the observability of a model error.

Model errors arising in kinetic reaction systems can originate from erroneous rate equations or lacking reactions. The dynamic elastic-net can detect both types of errors as hidden inputs to the corresponding nodes of the network, but it can not discriminate between these errors. However, knowing the nodes affected by a model error might already be very informative for systematic model improvement.

In view of the rapid progress of technologies to monitor biological dynamics, our approach could have implications for many fields including metabolic engineering, synthetic biology and and pharmacokinetics/pharmacodynamics. As our method is designed for generic ODE models, it can also be applied to challenging modelling tasks in engineering, robotics and in the earth sciences. Our work also raises fundamental questions regarding successful modelling strategies. The approach to manually include more and more details into the model to compensate the initial model errors is often not practical or at least very time consuming. The dynamic elastic-net hence paves the way towards a more principled and systematic way, in which models could be adapted based on experimental data.

## Methods

### Software

Simulations were performed in MATLAB (R2014a, The MathWorks, Inc.) using TOMLAB v8.0 with SQOPT 7.2–5 QP and SNOPT 7.2–5 NLP (Tomlab Optimization AB) for solving the optimal control problems. MATLAB scripts are provided as Supplementary material. The computing time for a single run of the dynamic elastic-net on a laptop (Intel CoreTM i5-4200M CPU with 4 × 2.50 GHz and 16 GB RAM) was between 3 seconds and 1 min.

### Data and models

Data for the JAK-STAT system^4^ were downloaded from http://webber.physik.uni-freiburg.de/~jeti/PNAS_Swameye_Data. Model equations for the UV-B signaling network^19^ were obtained from the Biomodels data base^3^, see BIOMD0000000545. For parameter values and mathematical details see the Supplementary text.

## Additional Information

**How to cite this article**: Engelhardt, B. *et al.* Learning (from) the errors of a systems biology model. *Sci. Rep.* **6**, 20772; doi: 10.1038/srep20772 (2016).

## Supplementary Material

Supplementary Information
