# Supplementary material for: Learning (from) the errors of a systems biology model
Source: Sci Rep. 2016 Feb 11;6:20772. doi: 10.1038/srep20772 (PMC4749970; doi:10.1038/srep20772)
Supplement: Supplementary Information [file srep20772-s1.pdf]

Supplemental text: Learning (from) the errors of a systems biology model

Benjamin Engelhardt[1], Holger Fröhlich[1] and Maik Kschischo[2]

[1]Rheinische Friedrich-Wilhelms-Universität Bonn, Institute for Computer Science, Algorithmic Bioinformatics, c/o Bonn-Aachen International Center for IT, Dahlmannstr. 2, 53113, Bonn, Germany
[2]University of Applied Sciences Koblenz, RheinAhrCampus, Department of Mathematics and Technology, Joseph-Rovan-Allee 2, 53424 Remagen, Germany

1 Analysis of the dynamic elastic-net optimal control problem

To gain a deeper understanding of the dynamic elastic-net we assume that the output $\boldsymbol{y}(t)$ of the underlying true system

$$\dot{\boldsymbol{x}}(t) = \tilde{\boldsymbol{f}}(\boldsymbol{x}(t), \boldsymbol{u}(t)) + \boldsymbol{w}(t) \tag{1a}$$
$$\boldsymbol{y}(t) = \boldsymbol{h}(\boldsymbol{x}(t)) \tag{1b}$$

with initial conditions $\boldsymbol{x}(0) = \boldsymbol{x}_0$ can be measured for all time points t in the interval $[0, T]$. Accordingly, the error functional (equation 3c in the main text) is replaced by the continuous time error functional

$$\mathcal{J} = \int_0^T \|\boldsymbol{y}(t) - \hat{\boldsymbol{y}}(t)\|^2_{Q(t)} + \alpha_1 \|\hat{\boldsymbol{w}}(t)\|_1 + \frac{\alpha_2}{2}\|\hat{\boldsymbol{w}}(t)\|^2 dt \tag{2a}$$
$$= \int_0^T \sum_{i=1}^m \sum_{j=1}^m \left[q_{ij}(t)\,(y_i(t) - \hat{y}_i(t))\,(y_j(t) - \hat{y}_j(t))\right] + \sum_{i=1}^m \left[\alpha_1 |\hat{w}_i(t)| + \frac{\alpha_2}{2}\hat{w}_i^2(t)\right] dt \tag{2b}$$

with the symmetric weighting matrix $Q(t) = (q_{ij}(t))$. The continuous time version will make the subsequent analysis more transparent without altering the main conclusions.

The dynamic elastic-net for continuous time measurements is the given as

$$\min_{\hat{\boldsymbol{w}}(t)} \mathcal{J}[\hat{\boldsymbol{w}}(t)] \qquad \text{subject to} \tag{2c}$$
$$\dot{\hat{\boldsymbol{x}}} = \tilde{\boldsymbol{f}}(\hat{\boldsymbol{x}}(t), \boldsymbol{u}(t)) + \hat{\boldsymbol{w}}(t) \tag{2d}$$
$$\hat{\boldsymbol{y}}(t) = \boldsymbol{h}(\hat{\boldsymbol{x}}(t)). \tag{2e}$$

For most practical cases, this optimal control problem [1, 2] can only be solved numerically [3]. However, the analysis of the necessary optimality conditions provides interesting insights. These conditions are formulated in terms of the Hamiltonian

$$\begin{aligned} \mathcal{H} &= \boldsymbol{\lambda}^T(t)\left[\hat{\boldsymbol{f}}\left(\hat{\boldsymbol{x}}(t), \boldsymbol{u}(t)\right) + \hat{\boldsymbol{w}}(t)\right] \\ &\quad + \|\boldsymbol{y}(t) - \boldsymbol{h}\left(\hat{\boldsymbol{x}}(t)\right)\|_Q^2 + \alpha_1 \|\hat{\boldsymbol{w}}(t)\|_1 + \frac{\alpha_2}{2}\|\hat{\boldsymbol{w}}(t)\|^2 . \end{aligned} \tag{3}$$

The new dynamic variable $\boldsymbol{\lambda} : [0, T] \mapsto \mathbb{R}^n$ is called the co-state or the adjoint state. It fulfills the adjoint equation [2, 3]

$$\begin{aligned} \dot{\boldsymbol{\lambda}}(t) &= -\left[\frac{\partial \mathcal{H}}{\partial \hat{\boldsymbol{x}}}\right]^T \\ &= -\left[\frac{\partial \boldsymbol{f}\left(\hat{\boldsymbol{x}}(t)\right)}{\partial \hat{\boldsymbol{x}}}\right]^T \boldsymbol{\lambda}(t) - 2\left[\frac{\partial \boldsymbol{h}\left(\hat{\boldsymbol{x}}(t)\right)}{\partial \hat{\boldsymbol{x}}}\right]^T Q(t)\left[\boldsymbol{y}(t) - \boldsymbol{h}(\hat{\boldsymbol{x}}(t))\right] . \end{aligned} \tag{4}$$

Without further conditions, the final co-state $\boldsymbol{\lambda}(T)$ equals zero. Alternatively, one can impose the condition that the fit of the dynamic elastic-net output $\hat{\boldsymbol{y}}(T)$ at final time T is not too far from the observed output $\boldsymbol{y}(T)$ by

$$\left(\boldsymbol{y}(T)) - \hat{\boldsymbol{y}}(T)\right)^T Q(T)\left(\boldsymbol{y}(T)) - \hat{\boldsymbol{y}}(T)\right) - \Delta_T < 0, \tag{5}$$

where Δ is a given tolerance. For this terminal constraint, we obtain the boundary condition for the co-state

$$\boldsymbol{\lambda}(T) = \boldsymbol{\mu}^T \frac{\partial}{\partial \hat{\boldsymbol{x}}}\left(\boldsymbol{y}(T)) - \hat{\boldsymbol{y}}(T)\right)^T Q(T)\left(\boldsymbol{y}(T)) - \hat{\boldsymbol{y}}(T)\right) \tag{6}$$

$$= 2\boldsymbol{\mu}^T\left[\frac{\partial \boldsymbol{h}\left(\hat{\boldsymbol{x}}(T)\right)}{\partial \hat{\boldsymbol{x}}}\right]^T Q(T)\left[\boldsymbol{y}(T) - \boldsymbol{h}(\hat{\boldsymbol{x}}(T))\right] , \tag{7}$$

which involves additional Lagrange-parameters $\boldsymbol{\mu}$ to be determined. If the initial condition $\boldsymbol{x}_0$ is uncertain, then on can add a condition analogous to (5).

Insights about the effect of the elastic-net regularisation come from Pontryagins minimum principle [1, 2, 3], which requires a minimisation of the Hamiltonian (3) with respect to $\hat{\boldsymbol{w}}$. To this end, we rewrite the Hamiltonian as

$$\mathcal{H} = \boldsymbol{\lambda}^T \hat{\boldsymbol{f}}\left(\hat{\boldsymbol{x}}(t), \boldsymbol{u}(t)\right) + \|\boldsymbol{y}(t) - \boldsymbol{h}\left(\hat{\boldsymbol{x}}\right)\|_{Q(t)}^2 + \sum_{i=1}^n \mathfrak{h}_i(\lambda_i, w_i) \tag{8a}$$

$$\mathfrak{h}_i(\lambda_i, w_i) = \lambda_i \hat{w}_i + \alpha_1 |\hat{w}_i| + \frac{\alpha_2}{2}\hat{w}_i^2 . \tag{8b}$$

Minimisation of $\mathcal{H}$ requires $\mathfrak{h}_i$ to be either zero or negative. From the derivative

$$\frac{\partial \mathfrak{h}_i}{\partial \hat{w}_i} = \lambda_i + \alpha_1 \operatorname{sign}(\hat{w}_i) + \alpha_2 \hat{w} \tag{9}$$

we find that the Hamiltonian $\mathcal{H}$ is minimised for

$$\hat{w}_i^* = \begin{cases} -\frac{\lambda_i - \alpha_1}{\alpha_2} & \text{if } \lambda_i > \alpha_1 \\ 0 & \text{if } \lambda_i \in [-\alpha_1, \alpha_1] \\ -\frac{\lambda_i + \alpha_1}{\alpha_2} & \text{if } \lambda_i < -\alpha_1 . \end{cases} \tag{10}$$

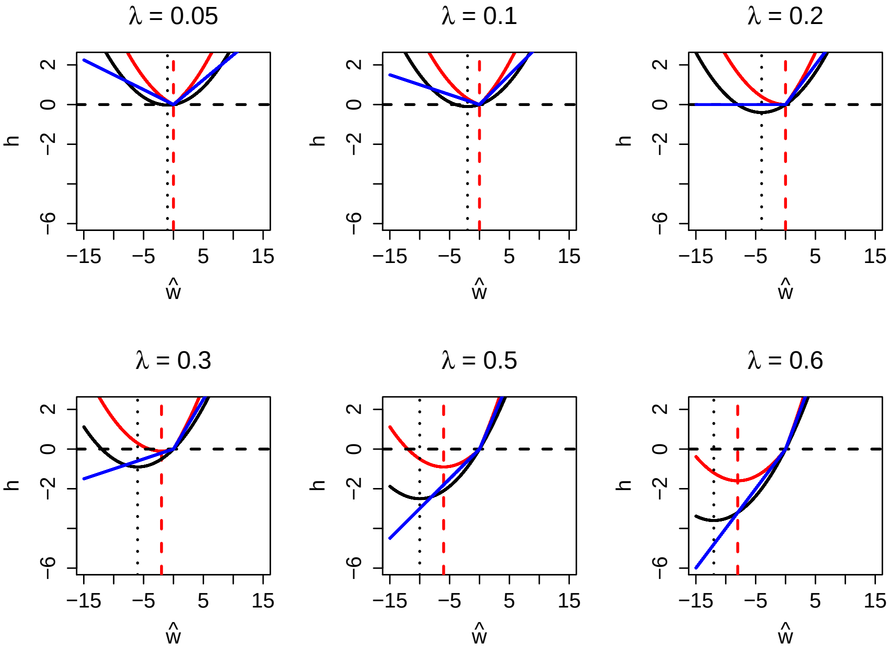

Figure 1: The function $\mathfrak{h}(\hat{w}) = \lambda\hat{w} + \alpha_1|\hat{w}| + \frac{\alpha_2}{2}\hat{w}^2$ (red) for different values of λ and $(\alpha_1, \alpha_2) = (0.2, 0.05)$. For comparison, the case of pure L_1 regularisation ($\alpha_2 = 0$, blue) and pure L_2 regularisation ($\alpha_1 = 0$, black) is shown. The dashed lines indicate the location of the minimum for the dynamic elastic-net (red) and for pure L_2 regularisation. The minimum of the pure L_1-case is always at zero or plus/minus infinity, if no further bounds are imposed on $\hat{w}$.

In Fig. 1 we compare the function $\mathfrak{h}_i$ and the location of the minimum w_i^* for the dynamic elastic-net to pure L_1 or L_2 regularisation. It can be seen, that a pure Lasso-type estimate ($\alpha_2 = 0$) is not useful, because the minimum will either be located at zero or at plus or minus infinity. Even when additional box constraints of $a \leq \hat{w}_i \leq b$ are imposed, the minimum can only jump between zero and the upper bound or zero and the lower bound [4].

2 An example for an unidentifiable model error

As an example of an unidentifiable model error we consider the following pair of linear systems

$$\begin{aligned} \dot{x}_1 &= -\alpha x_1 + x_2 + w_1(t) \\ \dot{x}_2 &= -\beta x_2 \\ y &= x_1 \end{aligned} \tag{11}$$

and

$$\begin{aligned} \dot{x}_1 &= -\alpha x_1 + x_2 \\ \dot{x}_2 &= -\beta x_2 + w_2(t) \\ y &= x_1 \end{aligned} \tag{12}$$

with the target points x_1 and x_2 respectively. Both systems are observable, as can be tested by the Kalman-condition. However, for any given input $w_2(t)$ and for

$$w_1(t) = \int_0^t e^{\beta\tau} w_2(\tau)\, d\tau \tag{13}$$

the output $y(t) = x_1(t)$ of both systems is identical. Thus, the model error is not observable.

3 Tuning the regularisation parameters

An important problem for regularised estimates is the choice of the tuning parameters, here α_1 and α_2 for L_1 and L_2 regularisation. In regression, these parameters are often chosen by cross validation or bootstrap [5]. The idea is to estimate the prediction error and to minimise this over a grid of α_1, α_2 values. This could in principle also be done for the dynamic elastic-net. However, the number of replicates of the time course measurements in systems biology is typically too small for resampling from the original data.

Another approach is given by the discrepancy method [6]. If the dynamic elastic-net output would not incur any bias, then the expectation $E(y_i(t) - \hat{y}_i(t))^2$ could be estimated by the variance σ_i^2 of the measurements. For a diagonal weighting matrix $Q = \mathrm{diag}(q_1, \ldots, q_m)$ we have for the variance of the square error term in (2a)

$$E\left(\sum_{i=1}^{m} q_i (y_i(t) - \hat{y}_i(t))^2\right) \approx \sum_{i=1}^{m} q_i \sigma_i^2 =: d\,, \tag{14}$$

provided that the residual measurement errors of the different output components are statistical independent and independent of time. Note, that for $q_i = \sigma_i^{-2}$ we have $d = m$. Thus, the right hand side d provides a first rough criterion to tune the regularisation parameters: Adjust α_1 and α_2 in such a way that the squared error term under the integral equals d. This can easily be extended to time dependent errors.

The sparsity of the dynamic elastic-net solution is controlled by α_1. We found empirically, that a good approach is to monitor the solution $\hat{\boldsymbol{w}} = (\hat{w}_1, \ldots \hat{w}_n)^T$ or the AUC values as a function of α_1. Typically, as α_1 is increased from zero, a sparse solution appears, which contains only a few dominant components. Further increasing α_1 does not change these components for a wide range of values, until the regularisation dominates the whole error functional (Fig. 2). We thus chose α_1 large enough to obtain a sparse solution and then tuned the L_2-parameter α_2 to obtain a good data fit using the discrepancy (14).

The regularisation parameters α_1 and α_2 as well as the weighting matrix Q used for the figures of the main text and for the additional examples below are collected in Tab. 1.

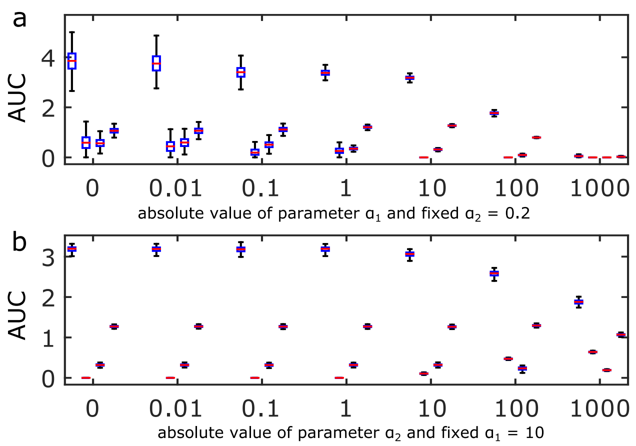

Figure 2: Influence of the regulation parameters for the JAK-STAT system. (a) The Box plots show the variation of the AUC of $|\hat{w}_1(t)|, ..., |\hat{w}_4(t)|$ for the dynamic elastic-net estimates caused by different values of the regulation parameter α_1 for $\alpha_2 = 0.2$. To ease visualisation, box plots for a given parameter value are slightly offset. (b) Influence of α_2 for fixed $\alpha_1 = 10$.

System	α_1	α_2	$Q \cdot N$	Figure
JAK-Stat	10	0.2	$\frac{1}{m}\text{diag}\left((\sigma_1^{-2}(t_k), \sigma_2^{-2}(t_k)\right)$	2, 3, S4, S5
JAK-Stat	1	0.2	$\frac{1}{m}\text{diag}\left(\sigma_1^{-2}(t_k), \sigma_2^{-2}(t_k)\right)$	S3
UV-B	0.1	0.0002	I	4, 5, S7, S8, S9
G protein	0.1	0.002	I	S10
FeedForwardLoop	0.01	0.002	I	S11
Diamond	0.1	0.002	I	S12
Bi-Fan	0.01	0.002	I	S13

Table 1: The regularisation parameters used in the main text. The standard deviation for output $y_i(t_k)$ at time point t_k is denoted as $\sigma_i(t_k)$. The number of measurement time points is N, the number of output components is m and I is a unit matrix.

4 Parameter values for the JAK-STAT model

The estimated parameters and their confidence intervals for the JAK-STAT system [7] were obtained from http://webber.physik.uni-freiburg.de/~jeti/PNAS_Swameye_Data and are shown in Table 2. Because of the structural non-identifiability of the parameters [8], the confidence interval of θ_2 was set to the range $[-3; 1]$. For the same reason, the confidence intervals of θ_5, θ_6 and $x_1(0)$ were not taken into account. Mass conservation was accounted for by the constraint $2x_4(t) + 2x_3(t) + x_1(t) + x_2(t) = \text{const.}$ for all t [7, 8].

	Value	CI
θ_1	+0.31	$[14; 48]$
θ_2	-1.00	$[-33; 31]$
θ_3	-0.49	$[-1.14; 0.15]$
θ_4	+0.42	$[0.28; 0.56]$
θ_5	-0.21	$[-32; 31]$
θ_6	-0.34	$[-32; 31]$
$x_1(0)$	+31	$[-32; 31]$

Table 2: Values of parameters and confidence intervals used for the JAK-STAT model (logarithmic scale).

5 Additional analysis of the JAK-STAT model

5.1 Comparison to the model of Raue *et al.* [8] to the thresholded dynamic elastic-net estimator

In addition to the analysis in the main text we compared the dynamic elastic-net to the model published in [8]

$$\begin{aligned}\dot{x}_1 &= -\theta_1 x_1 u + 2\theta_4 x_4^\tau \\ \dot{x}_2 &= \theta_1 x_1 u - 2\theta_2 x_2^2 \\ \dot{x}_3 &= \theta_2 x_2^2 - \theta_3 x_3 \\ \dot{x}_4 &= \theta_3 x_3 - \theta_4 x_4^\tau \\ \\ y_1 &= \theta_4 (x_2 + 2x_3) \\ y_2 &= \theta_5 (x_1 + x_2 + 2x_3).\end{aligned} \tag{15}$$

This model incorporates cytoplasmatic cycling by using the delay term $x_4^\tau := x_4(t - \tilde{\tau})$ with $\tilde{\tau} \approx 5$.

The estimates $\hat{\boldsymbol{w}}(t)$ described in the main text are dominated by $\hat{w}_1$ and $\hat{w}_4$. Thus, we fitted another dynamic elastic-net estimate enforcing $\hat{w}_2 = \hat{w}_3 = 0$. This thresholding [9] procedure can be used to decrease the bias caused by regularised least squares estimation. The delay term x_4^τ in model (15) is well represented by the scaled model error $\frac{K}{\theta_4}\hat{w}_4(t)$ of the thresholded estimate (Fig. 3a). The scaling factor $K = \frac{1400\mu m^3}{450\mu m^3}$ accounts for the volume ratio of the cytoplasmatic and nuclear compartements.

5.2 Taking the delay model as ground truth

In another test, we used the model (15) as ground truth and simulated data using the values of the standard deviation reported in [7]. We used the same nominal model as in the main text, which corresponds to a zero delay term $x_4^\tau = 0$. The dynamic elastic-net based on this nominal model correctly identifies the target points x_1 and x_4 (Fig. 4) and reconstructs the delay term $x_4^\tau(t)$ (Fig. 4g).

These results are also robust against measurement noise and parameter uncertainties (Fig. 5). The robustness simulations were performed as in the main

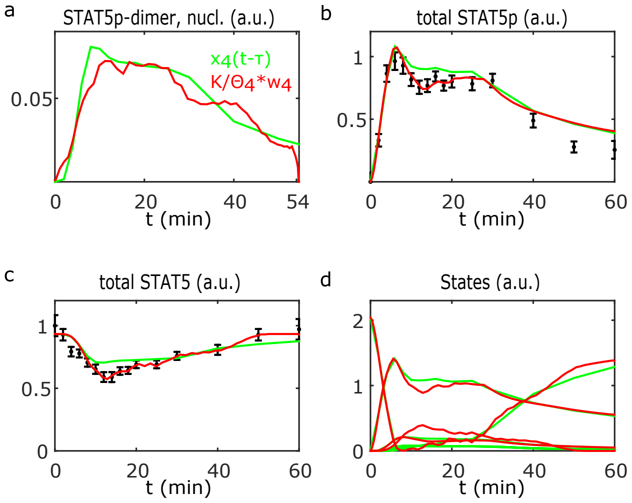

Figure 3: Comparison of thresholded dynamic elastic-net estimates (red) and the delay model (15) from [8] (green) with the real data (black). (a) The delay term x_4^τ representing cytoplasmatic cycling compared to the model error component $\hat{w}_4(t)$ of the thresholded dynamic elastic-net. The scaling factor K accounts for the volume ratio of the cytoplasmatic and nuclear compartements. The output of the delay model and the thresholded dynamic elastic-net compared to the measurements of total STAT5 (b) and total STAT5p (c). (d) Comparison of the state estimates.

text, but now we used the data simulated from the delay model (15) as observations.

5.3 Sensitivity against the number of measurements

To investigate the sensitivity of the dynamic elastic-net against the number of measurement time points we randomly chose subsets of the original data sets and run the optimisation using less input data. As it can be seen from Fig. 6, the AUC diagrams indicating the nodes targeted by model error are quite insensitive against a reduction of the number data points, as long as the main dynamic features of the time courses are still covered by the data.

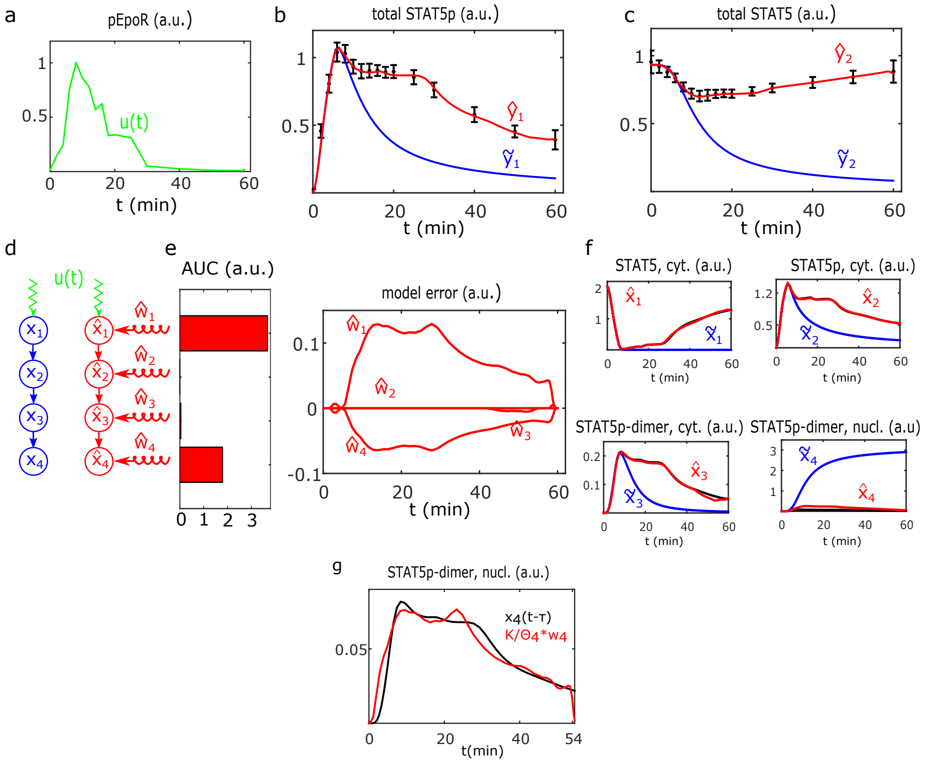

Figure 4: Reconstructing the model error for data simulated from the delay model (15) for the JAK-STAT pathway [8]. (a) As before, the known input $u(t)$ is given by linearly interpolated phosphorylation measurements for the erythropoietin receptor [7]. (b,c) The simulated output measurements (black) for phosphorylated cytoplasmatic STAT5 (y_1) and total cytoplasmatic STAT5 (y_2) compared to the outputs of the nominal model (blue) and the fit of the dynamic elastic-net (red). (d) Graph of the nominal model (blue) and of the observer system (red) with the state variables cytoplasmatic STAT5 (x_1), phosphorylated monomeric STAT5 (x_2), phosphorylated dimeric STAT5 (x_3) and nuclear STAT5 (x_4). (e) Dynamic elastic-net estimates of the model error. The area under the curve (AUC) indicating the target points of the model error estimates $\hat{w}_1(t), \ldots, \hat{w}_4(t)$. (f) The state estimates $\tilde{x}_1, \ldots \tilde{x}_4$ obtained from the nominal model (blue) and the dynamic elastic-net observer ($\hat{x}_1, \ldots \hat{x}_4$ in red) compared to the true state (black). (g) Comparison of the delay term (15) and the reconstruction by the dynamic elastic-net as already shown in (e).

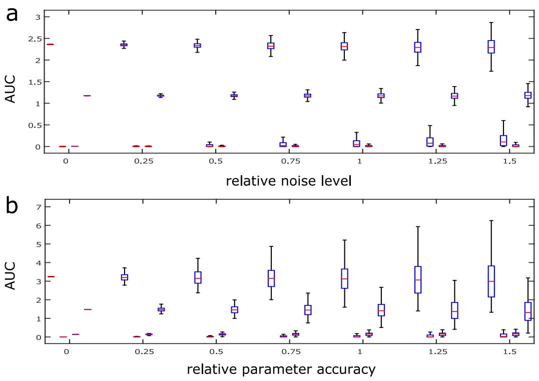

Figure 5: Robustness against measurement noise and parameter uncertainty using data simulated from the delay model (15). (a) Box plots visualising the variation of the AUC of the dynamic elastic-net estimates caused by measurement noise. To ease visualisation, box plots at a given noise level are slightly offset. (b) The variation of the AUC of caused by parameter uncertainty.

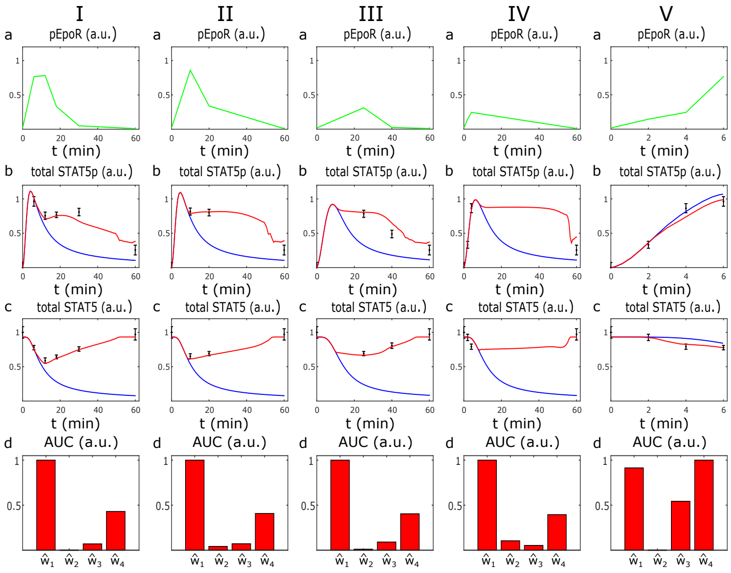

Figure 6: Sensitivity against the number and location of measurement time points for the JAK-STAT model [7, 8]. Four different subsets of data were used to fit the dynamic elastic-net (columns I to V). (a) The known input $u(t)$ is given by linearly interpolated phosphorylation measurements for the erythropoietin receptor [7, 8]. (b,c) The simulated output measurements (black) for phosphorylated cytoplasmatic STAT5 (y_1) and total cytoplasmatic STAT5 (y_2) compared to the outputs of the nominal model (blue) and the fit of the dynamic elastic-net (red). (d) The area under the curve (AUC) indicating the target points of the model error estimates $\hat{w}_1(t), \ldots, \hat{w}_4(t)$.

6 The photomorphogenic UV-B signaling network

The model equations (see section 8.7) for the UV-B signaling network [10] were obtained from the Biomodels data base [11], see BIOMD0000000545. The measurement noise of the simulated data in the main text was drawn from a Gaussian distribution with zero mean and standard deviation equal to 5% of the maximum value of the respective output signal.

6.1 Suboptimal solutions for the UV-B signaling network

Figs. 7 and 8 complement Fig. 5 in the main text to show that in cases, where the model error is not observable, a perfect fit of the output data can be obtained for different sets of target nodes.

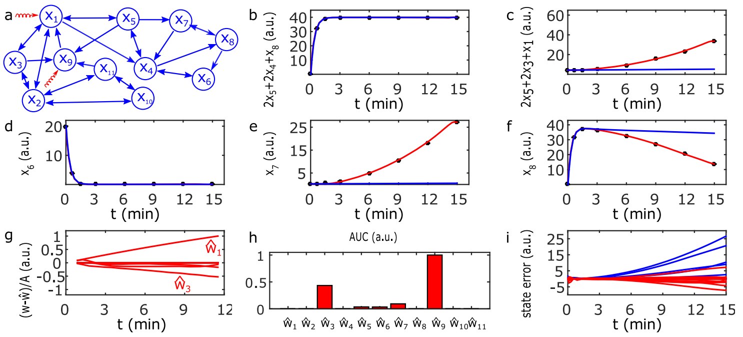

Figure 7: Dynamic elastic-net estimates for the photomorphogenic UV-B signaling network with model error targeting x_1 and x_9.

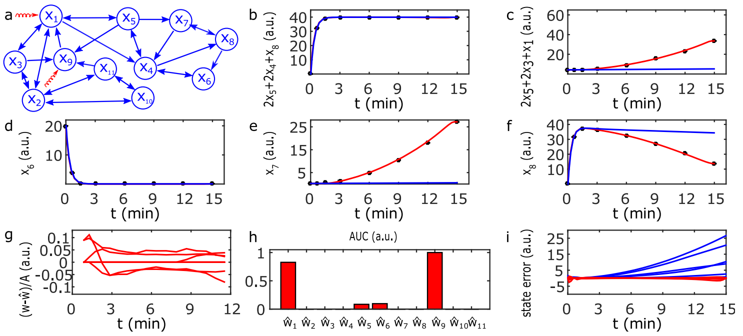

Figure 8: The corresponding estimate, when $\hat{w}_3$ is constrained to zero.

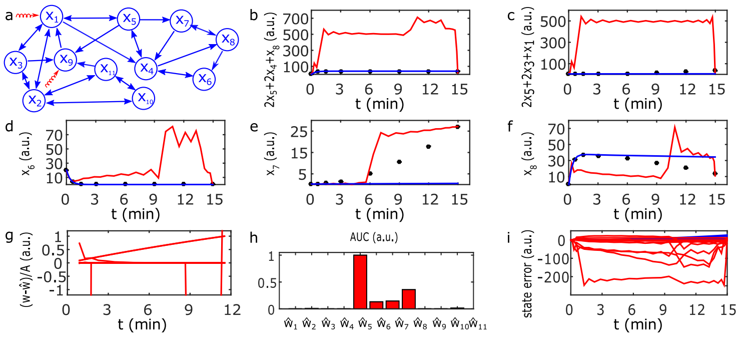

Figure 9: The corresponding estimate, when $\hat{w}_3$ and $\hat{w}_9$ are constrained to zero. The output data can not be fitted with this combination.

7 Additional examples

7.1 G protein signalling

A model for the heterotrimeric G protein cycle in yeast [12] was downloaded from the Biomodels data base [11] (BIOMD0000000072). The model has six state variables: The amount of receptor R, ligand bound receptor LR, inactive associated G protein $\overline{\mathrm{GP}}$ and its active subunits GP_α and $GP_{\beta\setminus\gamma}$ and an inactive subunit $\overline{GP}_\alpha$. We used this model as a nominal model, see section 8.6 for the equations and parameters.

The true model is assumed to have an additional input to x_4 representing $\overline{\mathrm{GP}}_\alpha$, which mimics a stronger regulation of GP_α. Synthetic measurement noise was added and the dynamic elastic-net was applied to reconstruct this model error (Fig. 10). All model states were assumed to be directly measurable.

7.2 Network Motifs

Network motifs are often considered to be building blocks of different biological networks [13]. Their dynamics will of course be influenced by inputs from their exogenous networks. We used three well studied network motifs (the feed-forward loop, the diamond and the bi-fan) to test, whether the dynamic elastic-net can reconstruct these hidden inputs from simulated data. We also investigated a phosphorylation cascade receptively feedback loop. Here, we assume that all state variables of the motifs are directly accessible to measurements. The model equations are given in sections 8.1-8.5. For all networks we used the unit step function

$$u(t) := 1 - \frac{1}{1+t}$$

as known input stimulus and the initial condition $\boldsymbol{x}(0) = \mathbf{0}$ for the state variables.

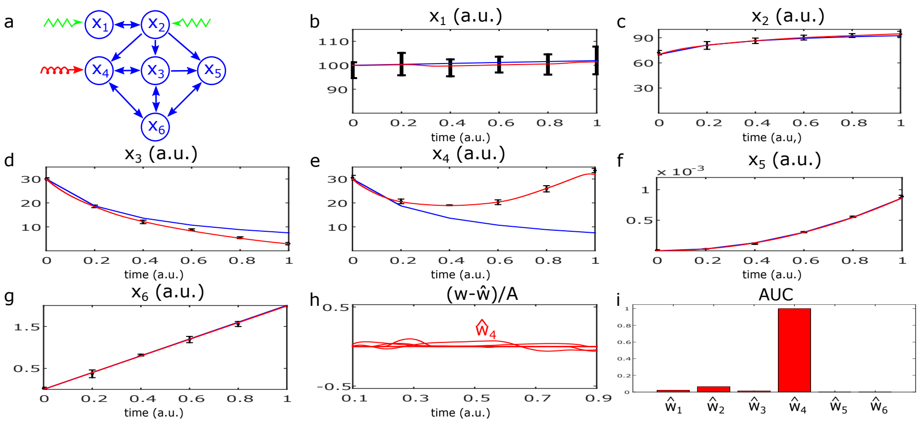

Figure 10: The heterotrimeric G protein cycle. (a) Graph of the nominal model with known inputs (green) and the additional hidden influence (red) (b)-(g) The simulated data for species x_i with its standard errors (black) compared to the nominal model (blue) and to the fit of the dynamic elastic-net (red). (h) The discrepancy between the true model error and the dynamic elastic-net estimate, scaled by the maximum value of the true model error. (i) AUC bars for the estimates of the model errors (in percent of total AUC).

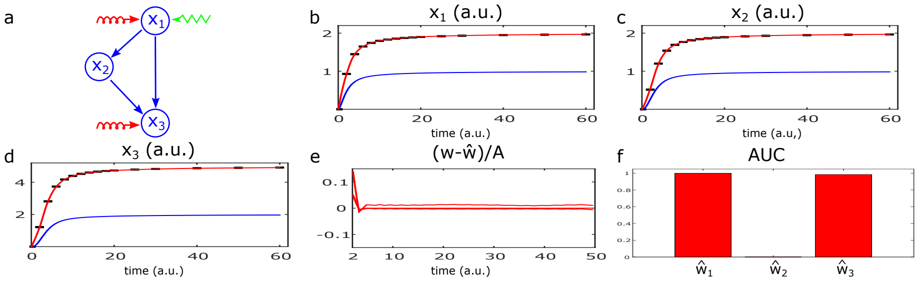

Figure 11: The feed-forward loop. (a) Graph of the nominal model with known inputs (green) and additional hidden influences (red) (b)-(d) The simulated data for variables x_i (black) compared to the nominal model (blue) and to the fit of the dynamic elastic-net (red). (e) Discrepancy between the dynamic elastic-net estimate $\hat{\boldsymbol{w}}$ and the true model error caused by hidden inputs from the environment, scaled by the maximum value of the true hidden input. (f) AUC for the dynamic elastic-net estimates.

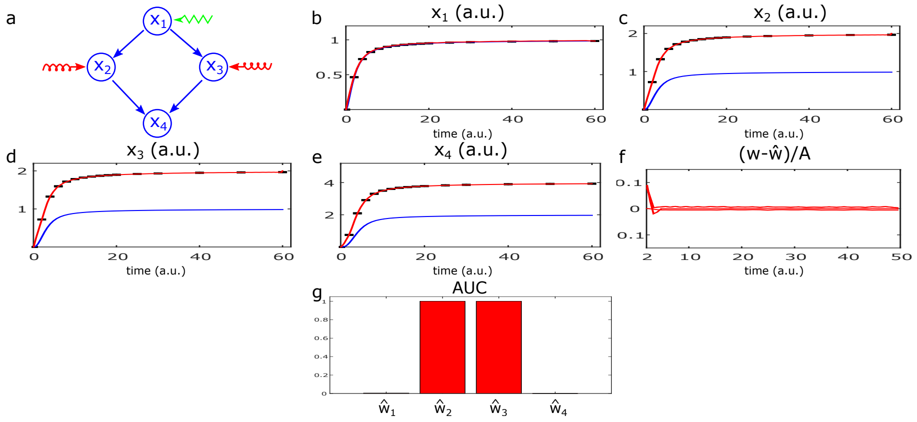

Figure 12: The diamond motif. (a) Graph of the nominal model with known inputs (green) and additional hidden inputs (red). (b)-(e) The simulated data for variables x_i (black) compared to the nominal model (blue) and to the fit of the dynamic elastic-net (red). (f) Discrepancy between the true error signal and the dynamic elastic-net estimate $\hat{\boldsymbol{w}}$, scaled by the maximum value of the true hidden input. (g) AUC for the dynamic elastic-net estimates.

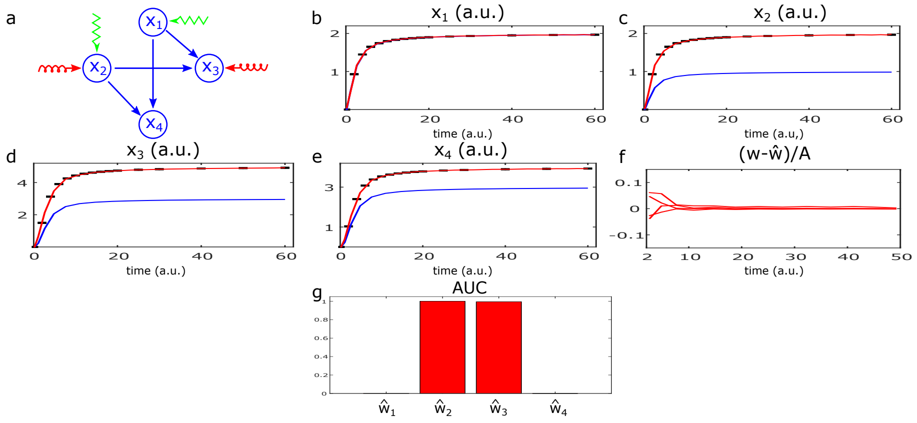

Figure 13: The bi-fan motif. (a) Graph of the nominal model with known inputs (green) and additional hidden influences (red) (b)-(e) The simulated data for variables x_i (black) compared for the nominal model (blue) and the fit of the dynamic elastic-net (red). (f) Discrepancy between the true error signal and the dynamic elastic-net estimate $\hat{\boldsymbol{w}}$, scaled by the maximum value of the true hidden input. (g) AUC for the dynamic elastic-net estimates.

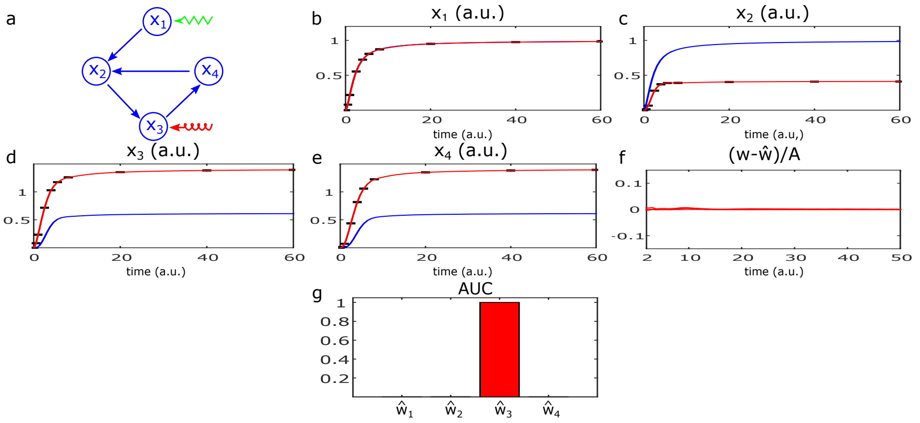

Figure 14: The feedback loop with exogenous error. (a) Graph of the nominal model with known inputs (green) and additional hidden influences (red) (b)-(e) The simulated data for variables x_i (black) compared for the nominal model (blue) and the fit of the dynamic elastic-net (red). (f) Discrepancy between the true error signal and the dynamic elastic-net estimate $\hat{\boldsymbol{w}}$, scaled by the maximum value of the true hidden input. (g) AUC for the dynamic elastic-net estimates.

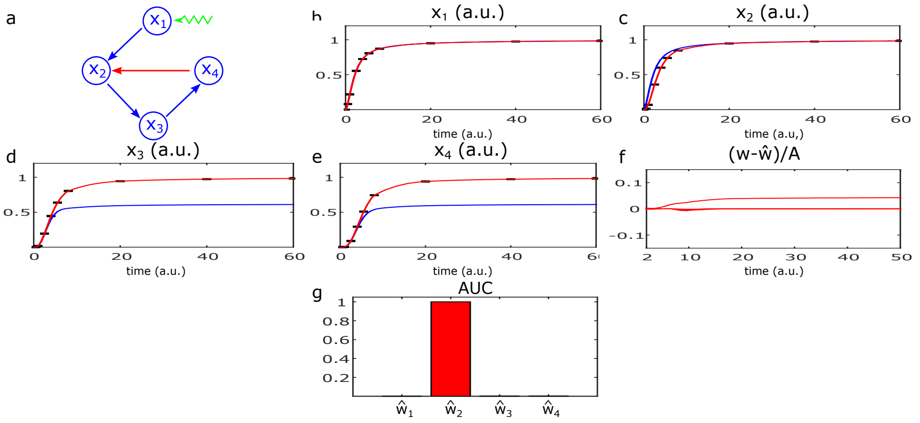

Figure 15: The feedback loop with endogenous error. (a) Graph of the nominal model with known inputs (green) and missing feedback (red) (b)-(e) The simulated data for variables x_i (black) compared for the nominal model (blue) and the fit of the dynamic elastic-net (red). (f) Discrepancy between the true error signal and the dynamic elastic-net estimate $\hat{\boldsymbol{w}}$, scaled by the maximum value of the true hidden input. (g) AUC for the dynamic elastic-net estimates.

8 Further model equations

8.1 Feed-forward Loop

True System

$$
\begin{aligned}
\dot{x}_1(t) &= u(t) - x_1(t) + \left(1 - \frac{1}{1+t}\right) \\
\dot{x}_2(t) &= x_1(t) - x_2(t) \\
\dot{x}_3(t) &= x_2(t) + x_1(t) - x_3(t) + \left(1 - \frac{1}{1+t}\right)
\end{aligned}
\tag{16}
$$

Nominal System

$$\begin{aligned}\dot{\tilde{x}}_1(t) &= u(t) - \tilde{x}_1(t)\\ \dot{\tilde{x}}_2(t) &= \tilde{x}_1(t) - \tilde{x}_2(t)\\ \dot{\tilde{x}}_3(t) &= \tilde{x}_2(t) + \tilde{x}_1(t) - \tilde{x}_3(t)\end{aligned} \tag{17}$$

8.2 Diamond

True System

$$\begin{aligned}\dot{x}_1(t) &= u(t) - x_1(t)\\ \dot{x}_2(t) &= x_1(t) - x_2(t) + \left(1 - \frac{1}{1+t}\right)\\ \dot{x}_3(t) &= x_1(t) - x_3(t) + \left(1 - \frac{1}{1+t}\right)\\ \dot{x}_4(t) &= x_2(t) + x_3(t) - x_4(t)\end{aligned} \tag{18}$$

Nominal System

$$\begin{aligned}\dot{\tilde{x}}_1(t) &= u(t) - \tilde{x}_1(t)\\ \dot{\tilde{x}}_2(t) &= \tilde{x}_1(t) - \tilde{x}_2(t)\\ \dot{\tilde{x}}_3(t) &= \tilde{x}_1(t) - \tilde{x}_3(t)\\ \dot{\tilde{x}}_4(t) &= \tilde{x}_2(t) + \tilde{x}_3(t) - \tilde{x}_4(t)\end{aligned} \tag{19}$$

8.3 Bi-fan

True System

$$\begin{aligned}\dot{x}_1(t) &= u(t) - x_1(t)\\ \dot{x}_2(t) &= u(t) - x_2(t) + \left(1 - \frac{1}{1+t}\right)\\ \dot{x}_3(t) &= \tilde{x}_2(t) + x_1(t) - x_3(t) - \left(1 - \frac{1}{1+t}\right)\\ \dot{x}_4(t) &= \tilde{x}_2(t) + x_1(t) - x_4(t)\end{aligned} \tag{20}$$

Nominal System

$$\begin{aligned}\dot{\tilde{x}}_1(t) &= u(t) - \tilde{x}_1(t)\\ \dot{\tilde{x}}_2(t) &= u(t) - \tilde{x}_2(t)\\ \dot{\tilde{x}}_3(t) &= \tilde{x}_2(t) + \tilde{x}_1(t) - \tilde{x}_3(t)\\ \dot{\tilde{x}}_4(t) &= \tilde{x}_2(t) + \tilde{x}_1(t) - \tilde{x}_4(t)\end{aligned} \tag{21}$$

8.4 Feedback Loop 1

True System

$$
\begin{aligned}
\dot{\tilde{x}}_1(t) &= u(t) - x_1(t) \\
\dot{\tilde{x}}_2(t) &= \frac{x_1(t)}{1 + x_4} - x_2(t) \\
\dot{\tilde{x}}_3(t) &= x_2(t) - x_3(t) + \left(1 - \frac{1}{1+t}\right) \\
\dot{\tilde{x}}_4(t) &= x_3(t) - x_4(t)
\end{aligned}
\tag{22}
$$

Nominal System

$$
\begin{aligned}
\dot{\tilde{x}}_1(t) &= u(t) - \tilde{x}_1(t) \\
\dot{\tilde{x}}_2(t) &= \frac{\tilde{x}_1(t)}{1 + \tilde{x}_4} - \tilde{x}_2(t) \\
\dot{\tilde{x}}_3(t) &= \tilde{x}_2(t) - \tilde{x}_3(t) \\
\dot{\tilde{x}}_4(t) &= \tilde{x}_3(t) - \tilde{x}_4(t)
\end{aligned}
\tag{23}
$$

8.5 Feedback Loop 2

True System

$$
\begin{aligned}
\dot{x}_1(t) &= u(t) - x_1(t) \\
\dot{x}_2(t) &= x_1 - x_2(t) \\
\dot{x}_3(t) &= x_2(t) - x_3(t) \\
\dot{\tilde{x}}_4(t) &= x_3(t) - x_4(t)
\end{aligned}
\tag{24}
$$

Nominal System

$$
\begin{aligned}
\dot{\tilde{x}}_1(t) &= u(t) - \tilde{x}_1(t) \\
\dot{\tilde{x}}_2(t) &= \frac{\tilde{x}_1(t)}{1 + \tilde{x}_4} - \tilde{x}_2(t) \\
\dot{\tilde{x}}_3(t) &= \tilde{x}_2(t) - \tilde{x}_3(t) \\
\dot{\tilde{x}}_4(t) &= \tilde{x}_3(t) - \tilde{x}_4(t)
\end{aligned}
\tag{25}
$$

8.6 G protein signalling model

The model for the heterotrimeric G protein cycle in yeast [12] was downloaded from the Biomodels data base [11] (BIOMD0000000072). This model was used as a nominal model in section 6 of this Supplementary text, but the tilde is suppressed for simplicity of notation.

$$
\begin{aligned}
\frac{\mathrm{d}([R])}{\mathrm{d}t} &= -\left(3.32e^{-18}\right)[L]_{const.}\,[\mathrm{R}] + 0.01\,[LR] - 4 - 0.0004\,[R] \\
\frac{\mathrm{d}\left(\left[\overline{\mathrm{GP}}\right]\right)}{\mathrm{d}t} &= \left[\overline{\mathrm{GP}}_{\alpha}\right]\left[GP_{\beta\backslash\gamma}\right] - \left(1e^{-05}\right)[LR]\left[\overline{\mathrm{GP}}\right] \\
\frac{\mathrm{d}\left(\left[GP_{\beta\backslash\gamma}\right]\right)}{\mathrm{d}t} &= -\left[\overline{\mathrm{GP}}_{\alpha}\right]\left[GP_{\beta\backslash\gamma}\right] + \left(1e^{-05}\right)[LR]\left[\overline{\mathrm{GP}}\right] \\
\frac{\mathrm{d}\left(\left[\overline{\mathrm{GP}}_{\alpha}\right]\right)}{\mathrm{d}t} &= -\left[\overline{\mathrm{GP}}_{\alpha}\right]\left[GP_{\beta\backslash\gamma}\right] + 0.11\,[\mathrm{GP}_{\alpha}] \\
\frac{\mathrm{d}([\mathrm{GP}_{\alpha}])}{\mathrm{d}t} &= \left(1e^{-05}\right)[LR]\left[\overline{\mathrm{GP}}\right] - 0.11\,[\mathrm{GP}_{\alpha}] \\
\frac{\mathrm{d}([LR])}{\mathrm{d}t} &= \left(3.32e^{-18}\right)[L]_{const.}\,[R] - 0.01\,[LR] - 0.004\,[LR]
\end{aligned}
\tag{26}
$$

These interactions are depicted in Fig. 10a. Initial conditions and the names of the state variables are given by

$$
\begin{aligned}
[R] &= x_1; & \left[\overline{\mathrm{GP}}_{\alpha}\right] &= x_4; \\
\left[\overline{\mathrm{GP}}\right] &= x_2; & [\mathrm{GP}_{\alpha}] &= x_5; \\
\left[GP_{\beta\backslash\gamma}\right] &= x_3; & [LR] &= x_6
\end{aligned}
$$

$$
\begin{aligned}
[R]_0 &= 100\ item\backslash l; & \left[\overline{\mathrm{GP}}_{\alpha}\right]_0 &= 30\ item\backslash l; \\
\left[\overline{\mathrm{GP}}\right]_0 &= 70\ item\backslash l; & [\mathrm{GP}_{\alpha}]_0 &= 0\ item\backslash l; \\
\left[GP_{\beta\backslash\gamma}\right]_0 &= 30\ item\backslash l; & [LR]_0 &= 0\ item\backslash l; \\
[L]_{const.} &= 0.02\ item\backslash l
\end{aligned}
$$

8.7 UVB Network model

The model equations (see section 8.7) for the UV-B signaling network [10] were obtained from the Biomodels data base [11], see BIOMD0000000545. Although this model was used as a nominal model in the main text, the tilde is suppressed for simplicity of notation.

$$\begin{aligned}
\frac{d\,[\mathrm{CS}]}{dt} =& -2 \cdot ka_1 \cdot [\mathrm{CS}]^2 \cdot [\mathrm{UVR8M}]^2 + 2kd_1 \cdot [\mathrm{UCS}] \\
&+ ks_1 \cdot (1 + UV \ \cdot \ n_3 \ \cdot \ ([\mathrm{HY5}] + FHY3)) \\
&- kdr_1 \cdot (1 + (n_1 \cdot UV)) \cdot [\mathrm{CS}] - kd_2 \cdot [\mathrm{CDCS}] \\
&- 2 \cdot ka_2 \cdot [\mathrm{CS}]^2 \cdot [\mathrm{CD}] \\
\frac{d\,[\mathrm{CD}]}{dt} =& - ka_2 \cdot [\mathrm{CS}]^2 \cdot [\mathrm{CD}] + kd_2 \cdot [\mathrm{CDCS}] \\
&+ ka_4 \cdot [\mathrm{CD}] \cdot [\mathrm{DWD}] + kd_4 \cdot [\mathrm{CDW}] \\
\frac{d\,[\mathrm{CDCS}]}{dt} =& - kd_2 \cdot [\mathrm{CDCS}] + ka_2 \cdot [\mathrm{CS}]^2 \cdot [\mathrm{CD}] \\
\frac{d\,[\mathrm{UVR8M}]}{dt} =& -2 \cdot k_1 \cdot [\mathrm{UVR8M}]^2 + 2 \cdot k_2 \cdot [\mathrm{UVR8D}] \\
&- 2 \cdot ka_1 \cdot [\mathrm{CS}]^2 \cdot [\mathrm{UVR8M}]^2 + 2 \cdot kd_1 \cdot [\mathrm{UCS}] \\
&- ka_3 \cdot [\mathrm{UVR8M}] \cdot [\mathrm{RUP}] \\
\frac{d\,[\mathrm{UCS}]}{dt} =& - kd_1 \cdot [\mathrm{UCS}] + ka_1 \cdot [\mathrm{CS}]^2 \cdot [\mathrm{UVR8M}]^2 \\
\frac{d\,[\mathrm{UVR8D}]}{dt} =& - k_2 \cdot [\mathrm{UVR8D}] + k_1 \cdot [\mathrm{UVR8M}]^2 + kd_3 \cdot [\mathrm{UR}]^2 \\
\frac{d\,[\mathrm{RUP}]}{dt} =& - ka_3 \cdot [\mathrm{UVR8M}] \cdot [\mathrm{RUP}] + ks_2 \cdot (1 + UV \cdot [\mathrm{UCS}]) \\
&- kdr2 \cdot [\mathrm{RUP}] + (2) \cdot kd_3 \cdot [\mathrm{UR}]^2 \\
\frac{d\,[\mathrm{UR}]}{dt} =& -2 \cdot kd_3 \cdot [\mathrm{UR}]^2 + ka_3 \cdot [\mathrm{UVR8M}] \cdot [\mathrm{RUP}] \\
\frac{d\,[\mathrm{HY5}]}{dt} =& - kdr3 \cdot \left(\frac{[\mathrm{CDCS}]}{kdr_{3a} + [\mathrm{CDCS}]} + \frac{[\mathrm{CDW}]}{kdr_{3b} + [\mathrm{CDW}]} \right) \cdot [\mathrm{HY5}] \\
&+ ks3_p \cdot (1 + n_2 \cdot UV) - kdr3 \cdot \left(\frac{[\mathrm{UCS}]}{ksr + [\mathrm{UCS}]} \right) \cdot [\mathrm{HY5}] \\
\frac{d\,[\mathrm{DWD}]}{dt} =& - ka_4 \cdot [\mathrm{CD}] \cdot [\mathrm{DWD}] + kd_4 \cdot [\mathrm{CDW}] \\
\frac{d\,[\mathrm{CDW}]}{dt} =& - kd_4 \cdot [\mathrm{CDW}] + ka_4 \cdot [\mathrm{CD}] \cdot [\mathrm{DWD}]
\end{aligned}$$

$$\begin{aligned}
UM_{Total} &= 2 \cdot [\mathrm{UCS}] + [\mathrm{UVR8M}] + [\mathrm{UR}] \\
COP1_{Total} &= 2 \cdot [\mathrm{UCS}] + 2 \cdot [\mathrm{CDCS}] + [\mathrm{CS}] \\
UVR8D_{obs.} &= [\mathrm{UVR8D}] \\
HY5_{obs.} &= [\mathrm{HY5}] \\
UVR8M_{obs} &= [\mathrm{UVR8M}]
\end{aligned}$$

$$[\mathrm{CS}] = x_1; \quad [\mathrm{UVR8M}] = x_4; \quad [\mathrm{RUP}] = x_7; \quad [\mathrm{DWD}] = x_{10};$$
$$[\mathrm{CD}] = x_2; \quad [\mathrm{UCS}] = x_5; \quad [\mathrm{UR}] = x_8; \quad [\mathrm{CDW}] = x_{11};$$
$$[\mathrm{CDCS}] = x_3; \quad [\mathrm{UVR8D}] = x_6; \quad [\mathrm{HY5}] = x_9$$

$$ks_1 = 0.23; \quad ks_2 = 4.0526; \quad k_1 = 0.0043;$$
$$kdr_1 = 0.1; \quad kdr_2 = 0.2118; \quad k_2 = 161.62;$$
$$ka_1 = 0.0372; \quad ka_2 = 0.0611; \quad ka_3 = 4.7207;$$
$$kd_2 = 50.6973; \quad kd_3 = 0.5508; \quad kd_1 = 94.3524;$$
$$ks_3 = 0.4397; \quad kdr_3 = 1.246; \quad UV = 1;$$
$$kd_4 = 1.1999; \quad n_1 = 3; \quad ka_4 = 10.1285;$$
$$n_2 = 2; \quad n_3 = 3.5; \quad kdr_{3a} = 0.9735;$$
$$ksr = 0.7537; \quad FHY3 = 5; \quad kdr_{3b} = 0.406$$

$$[\mathrm{CS}]_0 = 0.2mol \quad [\mathrm{RUP}]_0 = 0mol$$
$$[\mathrm{CD}]_0 = 10mol \quad [\mathrm{UR}]_0 = 0mol$$
$$[\mathrm{CDCS}]_0 = 2mol \quad [\mathrm{HY5}]_0 = 0.25mol$$
$$[\mathrm{UVR8M}]_0 = 0mol \quad [\mathrm{DWD}]_0 = 20mol$$
$$[\mathrm{UCS}]_0 = 0mol \quad [\mathrm{CDW}]_0 = 0mol$$
$$[\mathrm{UVR8D}]_0 = 20mol$$

References

[1] Pontryagin, L. S., Boltyanskii, V. G., Gamkrelidze, R. V. & Mishchenko, E. F. *The mathematical theory of optimal processes*. No. v. 1 in Classics of Soviet mathematics (Gordon and Breach Science Publishers, New York, 1986), english ed edn. 00000.

[2] Fleming, W. H. & Rishel, R. W. *Deterministic and stochastic optimal control*. Applications of mathematics ; 1 (Springer-Verlag, Berlin ; New York, 1975). 03009.

[3] Gerdts, M. *Optimal control of ODEs and DAEs*. De Gruyter textbook (De Gruyter, Berlin ; Boston, 2012). 00027.

[4] Vossen, G. & Maurer, H. OnL1-minimization in optimal control and applications to robotics. *Optimal Control Applications and Methods* **27**, 301–321 (2006). URL http://doi.wiley.com/10.1002/oca.781. 00000.

[5] Zou, H. & Hastie, T. Regularization and variable selection via the Elastic Net. *Journal of the Royal Statistical Society, Series B* **67**, 301–320 (2005). 03777.

[6] Honerkamp, J. & Schelter, B. O. *Statistical physics: an advanced approach with applications*. Graduate texts in physics (Springer, Berlin, 2014). 00000.

[7] Swameye, I., Müller, T. G., Timmer, J., Sandra, O. & Klingmüller, U. Identification of nucleocytoplasmic cycling as a remote sensor in cellular signaling by databased modeling. *Proceedings of the National Academy of Sciences* **100**, 1028–1033 (2003). URL http://www.pnas.org/content/100/3/1028.short. 00309.

[8] Raue, A. *et al.* Structural and practical identifiability analysis of partially observed dynamical models by exploiting the profile likelihood. *Bioinformatics* **25**, 1923–1929 (2009). URL http://dx.doi.org/10.1093/bioinformatics/btp358. 00253.

[9] van de Geer, S., Bühlmann, P. & Zhou, S. The adaptive and the thresholded Lasso for potentially misspecified models (and a lower bound for the Lasso). *Electronic Journal of Statistics* **5**, 688–749 (2011). URL http://projecteuclid.org/euclid.ejs/1311600467. 00047.

[10] Ouyang, X. *et al.* Coordinated photomorphogenic UV-B signaling network captured by mathematical modeling. *Proceedings of the National Academy of Sciences* **111**, 11539–11544 (2014). URL http://www.pnas.org/cgi/doi/10.1073/pnas.1412050111. 00003.

[11] Li, C. *et al.* BioModels Database: An enhanced, curated and annotated resource for published quantitative kinetic models. *BMC Systems Biology* **4**, 92 (2010). URL http://www.biomedcentral.com/1752-0509/4/92. 00322.

[12] Yi, T.-M., Kitano, H. & Simon, M. I. A quantitative characterization of the yeast heterotrimeric G protein cycle. *Proceedings of the National Academy of Sciences* **100**, 10764–10769 (2003). URL http://www.pnas.org/cgi/doi/10.1073/pnas.1834247100. 00155.

[13] Milo, R. Network Motifs: Simple Building Blocks of Complex Networks. *Science* **298**, 824–827 (2002). URL http://www.sciencemag.org/cgi/doi/10.1126/science.298.5594.824. 04094.
